# Analytical Methodologies for the Determination of Endocrine Disrupting Compounds in Biological and Environmental Samples

**DOI:** 10.1155/2013/674838

**Published:** 2013-05-08

**Authors:** Zoraida Sosa-Ferrera, Cristina Mahugo-Santana, José Juan Santana-Rodríguez

**Affiliations:** Departamento de Química, Universidad de Las Palmas de Gran Canaria, 35017 Las Palmas de Gran Canaria, Spain

## Abstract

Endocrine-disruptor compounds (EDCs) can mimic natural hormones and produce adverse effects in the endocrine functions by interacting with estrogen receptors. EDCs include both natural and synthetic chemicals, such as hormones, personal care products, surfactants, and flame retardants, among others. EDCs are characterised by their ubiquitous presence at trace-level concentrations and their wide diversity. Since the discovery of the adverse effects of these pollutants on wildlife and human health, analytical methods have been developed for their qualitative and quantitative determination. In particular, mass-based analytical methods show excellent sensitivity and precision for their quantification. This paper reviews recently published analytical methodologies for the sample preparation and for the determination of these compounds in different environmental and biological matrices by liquid chromatography coupled with mass spectrometry. The various sample preparation techniques are compared and discussed. In addition, recent developments and advances in this field are presented.

## 1. Introduction

**Figure 1:**
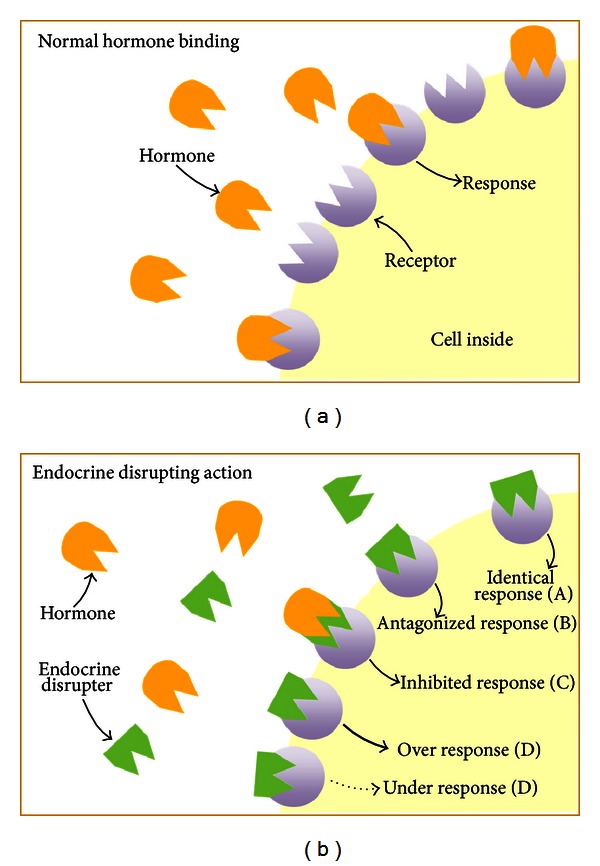
Scheme of the endocrine disrupting action: (A) mimic natural hormones, (B) antagonize their action, (C) alter their pattern of synthesis and metabolism, or (D) modify the expressions of specific receptors.

The global production of chemical products has increased in the last decades, and although many of the products have been beneficial for mankind, many of them are also toxic because they exhibit a long environmental persistence and can accumulate within organisms [1, 2]. Currently, many of the problems of pollution are due to intermittent spillage of these substances into the environment. In addition to their toxicity, persistence, and risk of bioaccumulation, these substances also clearly affect biological processes both in plants and in animals, including humans. The occurrence of chemical compounds that influence the sexual development of fish in English rivers was reported 15 years ago [3]. These exogenous substances that interfere with the endocrine system and disrupt the physiologic function of hormones are called endocrine-disrupting compounds (EDCs). The effects of natural and synthetic EDCs found in the environment include a decreased sperm count in human males and an increased risk of breast cancer and reproductive abnormalities in human females [4–6]. The endocrinal and reproductive effects of endocrine disrupting compounds may be a consequence of their ability to (a) mimic natural hormones, (b) antagonise their action, (c) alter their pattern of synthesis and metabolism, or (d) modify the expressions of specific receptors.  Figure 1 shows a scheme of the endocrine-disrupting action. 

Despite the increased interest in this type of pollutant that has arisen in the scientific community and the extensive work performed over the last two decades, important aspects, including the need to predict effects beyond the simply observed hormonal action that is implicated in the pathogeny of endocrine-related diseases, the level of exhibition of the general population, the identification of the threshold level of the effect, and the mechanisms of action and their adverse effects, have not been thoroughly investigated [7]. In conclusion, although a great deal of research related to the hazard assessment and regulation of EDCs has been published, a large number of uncertainties remain with respect to their actions. 

Many EDCs are not currently covered by existing regulations. A number of international organisations have made several attempts to establish a consensus related to EDCs; however, the number of families of so-called endocrine disrupting pollutants increases each year. The aim of this work is to provide the scientific community with a set of families of chemical compounds to which special attention should be devoted [8].

Although many natural and synthetic chemicals are widely considered to be EDCs, numerous chemicals present in the environment still remain unidentified and are considered potential EDCs. Moreover, many new chemicals are continually being produced in response to needs in various industrial sectors, and evidence of the endocrine disrupting compounds activities of some of these compounds is often controversial.

To date, several studies have demonstrated the negative effects of EDCs on wildlife and human health, which seem to occur even in cases of trace-level EDCs. Great variability, however, has been observed in chemical structures of EDCs that possess diverse characteristics with similar antiandrogenic and steroidogenesis activities [9–16]. Because of the large variety of suspected EDCs, humans and animals are most likely exposed not to a single agent, but rather to a mixture of multiple endocrine-disrupting agents.

**Figure 2:**
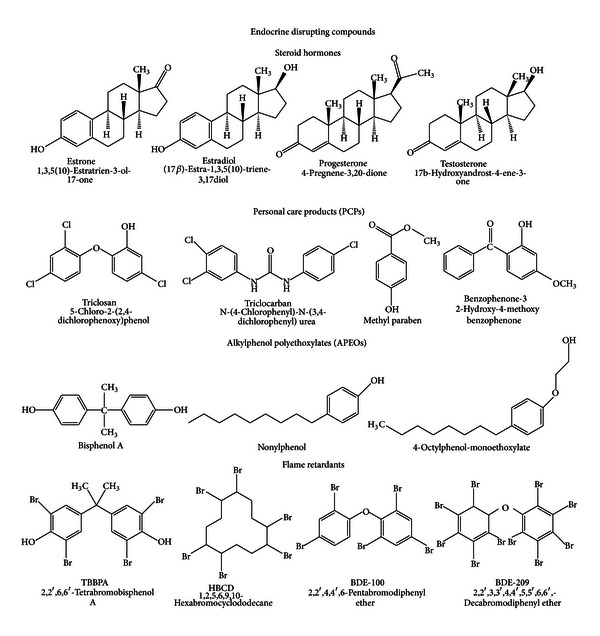
Chemical structure of EDCs more commonly found in environmental and biological samples.

Most EDCs are synthetic organic chemicals (xenobiotics) introduced into the environment by anthropogenic inputs; however, they can also be naturally generated estrogenic hormones (e.g., estrone or 17*β*-estradiol). Sources of EDCs include natural and synthetic hormones, personal-care products, pesticides, phthalates, alkylphenol ethoxylate surfactants, flame retardants, dioxins, coplanar polychlorinated biphenyls (PCBs), parabens, bisphenol A, and organotins [17].  Figure 2 shows the chemical structure of some of these compounds more commonly found in environmental and biological samples. A common characteristic to all of them is that they contain at least one aromatic moiety in their molecular structure. Thus, their hydrophobic properties might comprise an important characteristic of their behaviour. 

The fact that EDCs are ubiquitous in the environment, especially in aquatic ecosystems, has raised concern. Many researchers have shown that wastewater treatment plants (WWTPs) are major contributors to the presence of EDCs in the environment, where they enter via domestic and industrial discharges [18–20]. WWTPs achieve only partial removal of EDCs. As a consequence, these compounds have been found in the effluents from WWTPs, and they can therefore reach the surface and the groundwater. 

Because of the nonpolar and hydrophobic nature of many EDC, that they can be absorbed onto particulate materials. This behaviour suggests that the general effect of wastewater treatment processes should be to concentrate organic pollutants in the sewage sludge, whereas mechanical separation techniques, such as sedimentation, should result in significant removal of organic pollutants from the aqueous phase to primary and secondary sludges. As a result, the treated wastewater is discharged relatively free of EDCs; however, the EDCs are absorbed into sewage sludge, which could constitute a new source of pollution. The sludge from WWTPs can be applied to agricultural fields as a fertiliser. Current legislation regulates the agricultural use of sewage sludge based only on the concentration of toxic heavy metals and nutrients. However, following the measures that the European Commission (EC) began to implement in 1999, the third draft of a future Sludge Directive contained a proposal to place limits on several organic contaminants [21, 22].

The unmetabolised compounds present in the manure or their biologically active metabolites may move from the manure in the fields to the groundwater and eventually enter surface water, such as rivers and lakes, where they can affect aquatic organisms. They may persist in solid environmental matrices for a prolonged period. Their persistence depends on their photostability, binding and adsorption capacity, degradation rate, and leaching rate into the water. Their accumulation is due to their moderately high octanol-water partition coefficient (log⁡*K*_ow_ = 3–5) [23].

Depending on the EDC, their effects on biota have been observed at EDC concentrations as low as 0.1 ng·L^−1^ [20]. The determination of these chemicals is required to allow their environmental impact to be assessed. In recognition of these concerns, pressure to further develop advanced wastewater treatment methods, such as ozonation and activated carbon treatment for broad applications in municipal wastewater treatment, has increased in Europe.

In this context, we need to expand our knowledge about the occurrence, transport, and fate of all these contaminants in the environmental and in biological samples. Their analysis represents a difficult task because of the high complexity of the matrices analysed and because of the usually low concentration (ng·L^−1^) at which the target compounds are present in such samples. In addition, biotic samples are complex matrices that contain large amounts of possible interfering compounds that necessitate the use of extensive extraction and clean-up procedures to obtain extracts amenable to analysis. As a result, the reliable quantification of EDCs in both environmental and biological samples represents an enormous challenge to analytical chemists.

As a consequence, one of the major trends in analytical chemistry is the development of fast and efficient procedures for the trace analysis of target and nontarget organic compounds in complex matrices.

## 2. Advanced Instrumentation

In recent years, advances in instrumentation have resulted in significant progress in the detection of EDCs, the unambiguous identification of their structures, and the determination of their amount. A number of analytical methods have been reported in the literature for the analysis of the target compounds. High-performance liquid chromatography (HPLC) is the dominant modern analytical technique employed for the analysis of these types of compounds. The improvements achieved during the last few years in terms of sensitivity are mostly due to the development of hyphenated chromatography-mass spectrometry techniques, which are today the methods of choice for the determination of trace organic analytes in environmental and biological samples.

Mass spectrometry (MS) is the one of the most valuable detection techniques because it provides information of the molecular structure of the compounds and because it is highly sensitive and selective. The combination of chromatography and MS can separate a mixture into its individual components and subsequently analyse each compound in the mixture both qualitatively and quantitatively. For quantification, the selective-ion mode (SIM) of MS can be used to achieve high sensitivity. The huge interest in the application of LC-MS techniques has significantly stimulated developments and improvements in mass-analyser technology.

However, when highly complex matrices are investigated, triple quadrupole (QqQ) MS is required to improve the determination selectivity and the unequivocal identification of the target analytes [24]. These instruments, which used in tandem MS, are able to isolate the molecular ion of the compound of interest in the first stage of the mass analyser and obtain selective precursor-product ion transitions when operated in selected reaction monitoring (SRM) mode. The most intensive fragment ion from the precursor ion is used for quantification. A less sensitive secondary transition is used as the second criterion for confirmation purposes. QqQ features a wide linear range of at least three orders of magnitude. 

Recently, more advanced MS technologies, such as time-of-flight (TOF-MS) or linear ion trap (LIT-MS), have been introduced and represent a powerful new identification tool. New hybrid quadrupole-time-of-flight mass spectrometry (Qq-TOF-MS) allows the acquisition of full-scan product-ion spectra, which provide the accurate mass of the product ion. Based on the product-ion spectra, the structural elucidation of unknown compounds as well as the identification of target compounds can be achieved with a much greater degree of certainty [25].

At the same time, demand for high-throughput analysis is growing due to an increasing number of samples, and shortening of the analytical run times is often required. Three main modern approaches in HPLC methods enable the reduction of analytical time without compromising resolution and separation efficiency: the use of monolith columns, liquid chromatography conducted at high temperatures, and liquid chromatography at ultra-high pressures using columns packed with sub-2-micron particles [26, 27].

The use of a monolith-sorbent-based instead of porous-particle-based column packings has become popular in the field of bioanalytical applications over the past few years. Monoliths can accept high flow rates (up to 10 mL min^−1^) in conventional column lengths without generating high back-pressures, which is their main advantage. The efficiency and resolution of monolith sorbents are comparable to those of silica particles with a diameter of 3 *μ*m. Monolithic rods are prepared by sol-gel technology, which enables the formation of a highly porous material that contains both macropores and mesopores in its structure. Much greater flow rates can be used and the resolution of the monolith rod column is insignificantly less affected by particulate materials. In addition, the column back-pressure remains low. Another practical advantage is the short-time required for column equilibration when a mobile-phase gradient is used. However, monolith columns also suffer several drawbacks. One is the limited number of commercially available stationary phases (C8, C18, and plain silica only). Another is the internal diameters of monolith columns (i.e., 4.6 mm, 4.0 mm, and 100 *μ*m internal diameters are typical; however, 2.0 and 3.0 mm columns have not, as yet, been manufactured in all common column lengths). These two disadvantages reduce their application domains substantially. Large internal column diameters, which are more readily available in all column lengths, are not fully compatible with MS (mass spectrometry) and induce a high consumption of organic solvent, especially with flow rates as high as 10 mL·min^−1^. Finally, monoliths made of silica possess limited chemical stability (pH range 2–8) [26, 28, 29], which, again, limits their applicability.

Elevated temperatures in high-temperature liquid chromatography (HTLC) (*T* > 60°C) can also be used to perform rapid analysis using standard column lengths. In HTLC, in the viscosity of the mobile phase viscosity is reduced because of higher temperatures, which results in the method's primary advantage of faster analyses. The efficiency, the mass transfer, and the optimal velocity increase simultaneously with increasing temperature, which enables the use of high mobile-phase velocity. The low viscosity and the high diffusivity of a mobile phase at high temperatures produce much lower mass transfer resistance; in addition, organic solvent consumption may also be reduced, which is consistent with the principles of “green” chromatography. However, even with the aforementioned advantages, HTLC is not routinely used because it has some drawbacks. First, the limited availability of stable high-temperature-resistant packing materials is problematic. Second, unstable compounds may degrade [30, 31].

The fastest growing trend in chromatography continues to be the use of ultra-high-performance liquid chromatography (UHPLC). UHPLC uses short columns and small-diameter particles (sub, 2 *μ*m) in the stationary phase, which allows higher pressures and, ultimately, narrower LC peaks (5–10 *μ*s wide). In addition to providing narrow peaks and improved chromatographic separations, UHPLC dramatically shortens analysis times, often to 10 min or less [26, 27, 30–33]. Because of the very narrow peaks produced by UHPLC (i.e., peak widths on the order of a few seconds), the coupling of a UHPLC to an MS device with a rapid acquisition rate is critical to ensure high efficiency. 

The ion suppression/enhancement effects play an important role in LC-MS quantification, and the extent of these effects needs to be quantitatively assessed [34, 35]. The ion suppression and matrix effects can cause severe problems with the quantification in trace analyses. To eliminate any possible variations during the ionisation process and the mass analysis, such as the ion suppression/enhancement, the contamination of the ion source or the mobile phase, extraction losses, or any other unpredictable effects, an internal standard must be used. Because these matrix effects might detrimentally affect important method parameters (e.g., the limit of detection (LOD), the limit of quantification (LOQ), the linearity, the accuracy, or the precision), sample pre-treatments that involve isolation of the analytes, purification of the extracts, and preconcentration are required [36].

## 3. Sample Preparation

When developing analytical methods to determined EDCs, both the typically very low concentration at which these compounds occur in environmental and biological samples and the complex matrix composition should be taken into account. Both analyte isolation and preconcentration procedure are necessary to maximise the recovery of the analyte. In addition, EDCs concentration might fluctuate both in time and in space, and standards (e.g., deuterated one) and reference materials are not readily available. 

Sample preparation and clean-up are necessary for three main reasons: to remove interferences that would otherwise affect the determination of the analytes; to enrich the target compounds to detectable concentrations; and to perform solvent switching to the desired solvent conditions used for instrumental detection.

Samples obtained from biological materials are usually not directly compatible with HPLC analyses because of their complexity and protein content. Biological samples are problematic due to the irreversible adsorption of proteins in the stationary phase, which results in a substantial loss of column efficiency and an increase in backpressure [37]. 

**Figure 3:**
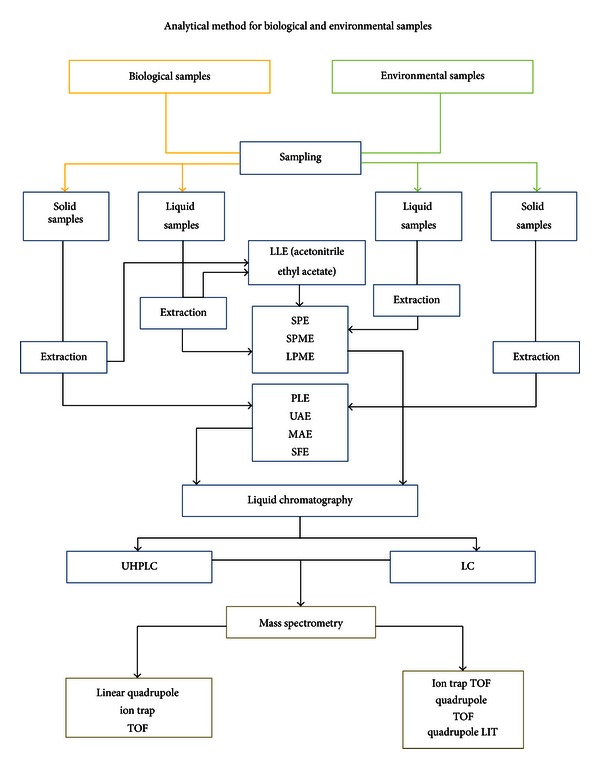
Scheme of the analytical method for the determination of EDCs in biological and environmental samples.

To obtain high recoveries and minimise interference, the determination of these pollutants requires extraction and clean-up steps prior to detection. The sample preparation steps often constitute the most time- and labour-intensive parts of the analytical process. A scheme that reflects the total analytical method for complex biological and environmental samples should be similar to that shown in Figure 3.

The sample extraction performed prior to instrumental analysis has several goals [38, 39]. Liquid-liquid extraction (LLE) and solid-phase extraction (SPE) are the traditional techniques used to extract organic compounds from liquid samples [40, 41]. SPE offers some advantages over LLE, such as improved selectivity, specificity and reproducibility, lower organic solvent consumption, shorter sample preparation time, easier operation, and the possibility of automation. In the SPE procedure, the choice of sorbent is critical because it controls selectivity, affinity, and capacity. Based on the characteristics of the target compounds, such as their polarity, and the sample matrix, different SPE sorbents can be chosen. SPE represents a broad field with numerous applications and has often been the subject of detailed studies and reviews [37, 41–45]. 

Much progress has been made recently toward improving adsorbent materials; the most relevant developments are advanced materials, such as restricted-access materials (RAMs) and molecularly imprinted polymers (MIPs). RAMs are designed specifically for the removal of macromolecules based on the size-exclusion mechanism, whereas MIPs are polymer-based materials that are formed during utilisation of the template molecules that play a role in target-compound recognition. Because of their selectivity, these materials have found applications primarily in areas where a large interfering substance is present in the complex matrix [41].

The development of online SPE configuration coupled with LC provides several advantages, including a reduction of the number of sample handling steps required, the elimination of the target loss by keeping the cartridge from drying, which results in an improved recovery and a reduction of the analysis time, and the minimisation of the volume of organic solvents consumed during each analysis. In general, online SPE-LC consists of a small precolumn placed in a six-port, high-pressure switching valve. During injection, the sample is preconcentrated on a precolumn with small dimensions to avoid band broadening in space; this column is pressure-resistant. The analytes are eluted onto the analytical column by valve switches [41].

 In multiresidue analyses, the greatest difficulty is the selection of the experimental conditions for the extraction. The optimisation of SPE conditions must lead to a compromise because the compounds exhibit different physicochemical properties. There are some disadvantages associated with SPE for determination of EDCs in environmental samples: SPE can be laborious and time consuming if the sample volumes are large (100–1000 mL per sample), and unwanted matrix components, which are typically present at much higher concentrations than the analytes of interest in matrices such as wastewater, coextract with the analytes. 

The demand to reduce the solvent volumes and avoid the use of toxic organic solvents has led to substantial efforts to adapt existing sample preparation methods to the development of new approaches. Consequently, during the last decade, researchers have made progress towards the development of more efficient extraction and clean-up techniques; the most recent tendencies have been towards automation through coupling of sample preparation units and detection systems; the application of advanced sorbents; and the application of greener approaches, such as reduced-solvent techniques. Miniaturisation has been a key factor in the approaches developed to satisfy these objectives. Microextraction techniques allow high-enrichment factors and minimise solvent consumption, which avoids environmental pollution. These techniques include solid-phase microextraction (SPME), stir-bar sorptive extraction (SBSE), and liquid-phase microextraction (LPME) approaches for liquid samples [38]. 

SPME is a modern equilibrium extraction method. A fused-silica fibre coated with a polymeric phase is usually utilised in SPME [46]. Typically, SPME method development requires optimisation of the equilibration conditions for each compound, which can make the development more difficult. SPME has shown some advantages over SPE: the sample volume is decreased; the procedure is simple because it incorporates sampling, extraction, concentration, and sample introduction into a single step; and individual fibres can be used for multiple extractions. However, SPME also still exhibits limitations, such as the short fibre lifetime, the high cost, fragility, and the occurrence of carry-over effects. Furthermore, it lacks selectivity in the extraction of analytes in complex matrices [47–50]. 

In an automated version of SPME, intube SPME, an open tubular fused-silica capillary with an inner surface coating, has been used as the extraction device; this technique is simple and can easily be coupled online with HPLC, [51, 52]. Analytes in liquid samples are directly extracted and concentrated onto the stationary phase by repeated draw/eject cycles or static sorption of the sample solution. The automation of the extraction process reduces the analysis time compared to that required for SPE and can also provide better accuracy, precision, and sensitivity than offline manual techniques.

This technique can overcome problems related to the use of conventional fibre SPME, such as fragility, low sorption capacity, and bleeding of thick-film coatings of the fibres. The main disadvantage of this technique is that it requires very clean samples because the capillary is easily blocked and the sample purification requires special instrumentation and operator experience.

Baltussen et al. [53] introduced a new and improved sample preparation technique based on the same principles as SPME: stir-bar sorptive extraction (SBSE). These stir bars, called Twisters (GERSTEL), are coated with a polydimethylsiloxane (PDMS) layer, which is the most widely used sorptive extraction phase. Although the basic principles of SPME and SBSE are generally identical and use the same extraction phase, the amount of PDMS is 50–250 times greater than that used in SBSE. This feature allows the preconcentration efficiency to be improved compared with SPME, which is the main advantage of SBSE [54, 55].

Although SPME has been the technique most widely used, in recent years, liquid-phase microextraction (LPME) approaches have attracted increasing interest. LPME can be considered a miniaturised form of LLE and overcomes many of its disadvantages while requiring minimal amounts of solvent [38, 56]. In this procedure, only a small amount of organic solvent is used for the extraction from an aqueous phase that contains the analytes of interest. LPME is simple to use, is generally rapid, and is characterised by its affordability and reliance on widely available materials [38]. Research on this technique began with researchers who used small droplets of organic solvents suspended from the tip of a microsyringe needle. However, new approaches have been developed to analyse compounds of a different nature and to achieve large enrichment factors using relatively short extraction times [38, 56].

**Figure 4:**
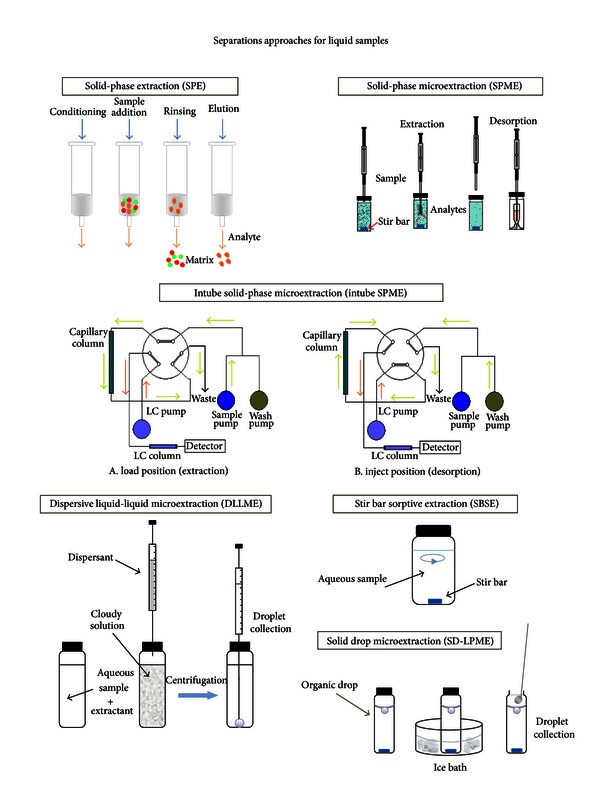
Schematic illustrations of separation approaches for biological and environmental samples.

Basic schematic illustrations of the principles of the separation approaches used for biological and environmental liquid samples are shown in Figure 4. 

As previously mentioned, due to the high octanol-water partition coefficient and the low biodegradability of many EDCs, they could be found bound to sewage sludge and or tissue samples. To date, most of the reported analytical methods for the determination of these selected contaminants in the environment have been focused on aqueous matrices (e.g., surface water and sewage water). Numerous methodologies have been developed for analysis of such contaminants in solid matrices, with sediments having been investigated slightly more than sewage sludge, most likely because of the complexity of the latter matrix. In addition to the pollutants of interest, sewage sludge contains a number of other components that potentially interfere in the analysis of the pollutants of interest; their removal from the sample using an established extraction and clean-up procedure is therefore critical.

For the analysis of biological samples, the samples are generally wrung and stored at −18°C before analysis. After the samples have been spiked at the desired level, they are mixed and homogenised in an organic solvent, such as acetonitrile, and then sonicated and centrifuged. 

In recent years, extraction methods have usually been based on liquid partitioning with ultrasonic extraction (USE), microwave-assisted extraction (MAE) or with the more advanced techniques of pressurised liquid extraction (PLE), or supercritical fluid extraction (SFE), which have replaced Soxhlet extraction [57, 58]. These techniques offer important benefits, such as a short extraction time, decreased solvent consumption, and decreased sample handling, in comparison to the traditional Soxhlet extraction procedure. The most effective method for the clean-up of extracts of solid samples that contain EDCs residues has proved to be solid-phase extraction (SPE).

Ultrasound-assisted extraction (UAE) involves the application of ultrasound radiation to the samples in a water bath or using other devices [59–61]. The typical extractants are methanol, ethanol, acetonitrile, and acetone in the mL range of volume, and the sonication times lie in the range of 2–120 min. After sonication, the extracted analytes are separated from the matrix by vacuum filtration or centrifugation. The process is repeated two or three times to achieve higher extraction efficiencies; the extracts are then recombined, and a clean-up procedure with SPE cartridges is applied prior to the analysis. The main disadvantage of UAE is poor reproducibility because of the lack of uniformity in the distribution of ultrasound energy, together with low selectivity and limited sample-enrichment capabilities. UAE is not easily automated and is not suitable for volatile analytes. A risk in the application of UAE to organics is the potential degradation of the analytes that may occur upon sonication [62]. A number of papers have been published dealing with ultrasound-assisted extraction of various EDC residues [63–67].

Microwave-assisted extraction (MAE) can be used to improve the efficiency of the extraction process. In MAE, microwave energy is used to heat solvents in contact with solid samples and to partition analytes from the sample matrix into the solvent (the extractant). In principle, only samples or solvents that contain dipolar materials or microwave absorbents are affected by microwaves. Since its initial development, MAE has become a byble alternative to conventional methods because it offers substantial improvements over other sample-preparation techniques: for example, shorter extraction times (the solvent is heated rapidly; an average extraction takes 15–30 min), the use of smaller amounts of solvent (between 10–30 mL), and increased sample throughput (multiple samples can be extracted simultaneously) [68]. The temperature, the extraction time and power, the solvent volume, and the concentration of different solvent mixtures are the most common parameters to be optimised. 

The number of papers that have reported the use of MAE has therefore increased considerably [69–72]. MAE systems can operate in two modes: open (focused MAE) or closed (pressurised MAE) vessels. In the latter devices, the solvent is heated and pressurised. Additional clean-up of the extract of the samples is generally necessary prior to analysis, and MAE is not amenable to automation (online extraction and detection).

The main advantage of MAE is most likely its wide applicability for the fast extraction of analytes, including the extraction of EDCs from soils and sediments [73–76] and from animal tissues [77, 78]. Although some examples of hyphenated MAE-based systems have been described [79, 80], the difficulty in integrating an MAE device into a flow system represents one of the main shortcomings of this technique.

Pressurised liquid extraction (PLE), also called accelerated solvent extraction (ASE), is a new extraction technique that provides the opportunity to reduce the extraction time and the solvent consumption (15–30 mL) with a high level of automation and to obtain better recoveries than those achieved with the classical extraction techniques through the use of higher temperature and/or higher pressures [70, 81, 82]. These aspects result in an increased efficiency and an increased rate of extraction. In a conventional PLE, the sample, which is typically dispersed in a drying or inert sorbent, such as sodium sulphate, hydromatrix, or diatomaceous earth, is packed into a stainless-steel cell and, after the cell has been inserted into a closed flow-through system, the sample is extracted with the selected solvent at temperatures greater than its atmospheric boiling point. This technique offers the advantage that only two variables need to be optimised: the extraction time and the temperature. Moreover, PLE provides cleaner extracts than Soxhlet and ultrasonic extraction, which results in reduced background noise during the subsequent determination; the reduction of background noise is especially important in LC-MS analysis due to ion-suppression effects. The main limitations of PLE are that the selectivity towards the analytes during extraction is not as high as might be desired and that many interferents may be coextracted, depending on the type of sample. Other disadvantages include dilution of the analytes, especially when a large number of cycles are used [83], and the high initial cost of these extraction systems. PLE is a well-established technique and has been used for the extraction of a wide variety of compounds from numerous matrices [84–88]. 

To a lesser extent, supercritical-fluid extraction (SFE) has been used for the extraction of EDCs from solid samples and has been reported to exhibit several advantages (e.g., rapid extraction, low solvent requirement, low cost, and higher efficiencies). SFE can be mainly applied for the extraction of nonpolar and slightly polar analytes. Among all the solvents used in SFE, pure CO_2_ is the most popular because of its low critical properties, chemical inertness, low toxicity and cost, and its ability to dissolve a wide range of organic compounds, including those with high molecular masses. Nevertheless, pure CO_2_ leads to low recoveries for polar compounds. The lack of extraction efficiency for polar compounds can be overcome through the addition of modifiers or cosolvents to the pure CO_2_, with water and methanol being the most commonly used solvent modifiers.

 The properties of supercritical fluids are intermediate between those of gases and liquids and depend on the pressure, temperature, and composition of the fluids. Their viscosity is lower than that of liquids, and their diffusion coefficients are higher, which leads to more efficient extractions [89]. A SFE system consists of a high-pressure pump that delivers the fluid and an extraction cell in which the sample is maintained at the correct pressure and temperature. The extracted analytes are trapped in an organic solvent or on a solid phase (the analytes are later eluted using an organic solvent). Parameters such as the temperature, the pressure, the extraction time, and the collection solvent are optimised. The high rate of penetration of the supercritical fluid in the sample permits the rapid diffusion of analytes, which reduces the extraction time. The complete process is performed in less than 20 or 30 min instead of the several hours required with other techniques. An advantage of SFE is that the extracts are very clean and require only moderate additional cleanup. However, the small volume of the extractor, which accommodates only a few grams of material, is a disadvantage when a larger sample mass is required. The principles and applications of this technique for solid matrixes and for different types of compounds have been reported [90–93].

## 4. Endocrine Disrupting Compounds

There is growing alarm over the potential adverse effects of environmental contaminants, such as those involving the endocrine functions. In this respect, we focused our attention on some contaminants of particular concern that exhibit varying degrees of endocrine-disrupting properties. In particular, we concentrated on four representative groups of compounds that differ in their nature and origin: natural and synthetic hormones, personal-care products, alkylphenol ethoxylate and bisphenol A, and brominated flame retardants. 

### 4.1. Hormones

Hormone residues constitute a recognised group of emerging environmental contaminants that have been proven to affect the biological activity of organisms exposed to them. The hormones interfere with endocrine function, and their presence in the environment has been observed to produce estrogenic effects, such as fish feminisation, changes in reproduction and behaviour, a decrease in the amount of spermatozoids, increased incidences of breast cancer in women, and increases in certain anomalies in the human reproductive system, even at low concentrations [10, 94, 95]. Thus, the determination of the fate and distribution of steroids and steroid conjugates in the environment is important because these compounds are potential sources of active estrogens as a result of dissociation in sewage treatment plants or the input of treated wastewater directly into surface waters. 

Steroid hormones (estrogens, progestogens, androgens, and corticosteroids) are considered the most potent active EDCs present in the environment; they are formed naturally by humans and wildlife and are produced synthetically. Therefore, interest in the sensitive determination of steroids in biological and environmental samples has increased in recent years. The trace-level determination of these compounds with similar structures contained in complex sample matrices requires the development of analytical methods with high sensitivity, selectivity, and resolution. These methods have been applied to soil, sediment, water, and other environmental samples, such as biological samples [96]. 

Kuster et al. [97] developed a method for investigating the presence of 21 emerging contaminants of various chemical groups (including seven estrogens and three progestogens) in the Llobregat river basin (Spain). They used SPE as an extraction method followed by LC-MS^2^ analysis. The method detection limits were less than or equal to 0.85 ng·L^−1^ for estrogens and less than or equal to 3.94 ng·L^−1^ for progestogens. Of the estrogens and progestogens analysed, only estrone-3-sulfate, estrone, estriol, and progesterone were found to be present in the low nanogram-per-litre range in some of the samples investigated. Miège et al. studied estrogenic disrupting potency in rivers and wastewaters in the Orge catchment area near Paris; they used LC-MS^2^ that was used for the determinations of natural estrogens and synthetic estrogens (ethinylestradiol) [98]. The estrone in all samples was in the range of 0.1–15.7 ng·L^−1^, whereas *β*-estradiol was measured at a lower concentration level (0.1–2.3 ng·L^−1^). No *α*-estradiol was detected. Ethinylestradiol was only detected in WWTP effluent at 0.2 ng·L^−1^, whereas estriol was detected in WWTP effluent at 12.1 ng·L^−1^ and in downstream effluent at 4.9 ng·L^−1^.

Chang and Huang [99] developed a method for the simultaneous determination of eighteen androgens and progestogens in environmental waters using UHPLC-MS^2^. The developed method was applied to the analysis of these compounds in wastewater and surface-water samples, and LODs for the eighteen analytes in the influent, effluent, and surface-water samples were in the ranges of 0.20–50, 0.04–20, and 0.01–12 ng·L^−1^, respectively.

The concentrations of several estradiol-mimicking compounds, including 17*β*-estradiol, estriol, and 17*α*-ethynylestradiol, in sewage sludge samples were determined using MAE followed by LC-MS^2^ with ESI in positive mode. The method provided LODs that ranged from 0.6 to 3.5 *μ*g·kg^−1^ [75]. A greater group of steroids, including natural and synthetic estrogens, androgens, progestogens and glucocorticoids, were determined in the same type of sample through the use of UAE followed by analysis by rapid-resolution LC-MS^2^. In this case, the LODs for the 28 analytes were 0.08–2.06 *μ*g·kg^−1^ [100]. LC-ESI(PI)-MS^2^ and PLE were used as extraction technique to determine traces of steroid hormones (including estrogens, androgens, and progestogens) in soil; the LODs were in the range 0.08–0.89 *μ*g·kg^−1^. The results obtained showed ionisation suppression for all of the analytes in proportions that ranged to almost 50% [88].

Steroid compounds in biological samples are difficult to analyse because of the broad range of substances, the complexity of the matrices, and the low levels of detection that must be achieved. As a result, a highly specific extraction technique is not feasible. MeOH is the most widely used solvent for the extraction of steroids from tissue samples. Blasco et al. described a procedure for the isolation of 22 steroids residues from bovine, pork, and poultry muscle tissues using MeOH. The crude extract was cleaned up by solid-phase extraction (SPE) using C18 and NH2 columns followed by an LC/ESI-MS^2^ method. However, the method development activities in this study indicated that ACN was a more selective extraction solvent [101]. In urine, steroids can be present in free forms, that is, as glucuronic acid and sulphate forms, which necessitates the inclusion of enzymatic hydrolysis to liberate the conjugates. Shao et al. have reported that the portion of cleavable conjugated forms of steroids in tissue are very low, which calls into the question the requirement for a deconjugation step [102].

More advanced techniques for the extraction of steroids hormones from matrices of animal origin have been reported; however, these techniques are far less common than the classical techniques previously described. Hooijerink et al. described a method for isolating six gestagens from kidney fat using accelerated solvent extraction (ASE) [103]. Han et al. extracted 17*α*-methyltestosterone in aquatic products using 1,1,1,2-tetrafluoroethane (R134a) as a subcritical fluid [104].

Table 1 shows diverse determinations of these compounds in biological and environmental samples.

### 4.2. Personal-Care Products

Personal-care products (PCPs) are the focus of much research because of their potential impacts on human and environmental health and because of increased public awareness and concern. Despite the growing availability of PCPs environmental occurrence data, few studies have directly addressed the potential human health relevance of these and other nonregulated xenobiotics in wastewater and wastewater-affected receiving waters. Even fewer studies have directly addressed the potential ecological impacts. Through the use of PCPs and other household products, humans are continually exposed to synthetic musks, preservatives and antimicrobials, sunscreen filters, and insect repellents. Dermal contact can be a major route of exposure to these compounds. They are lipophilic and persistent in the body; therefore, they are expected to accumulate in lipid-rich tissues, human milk, and blood. Some studies have suggested that the half-lives of these compounds are on the order of several months [140]. Numerous studies have reported PCPs occurrence data and have established that PCPs are ubiquitous in wastewater treatment plant effluents [141–145]. 

Table 2 shows diverse determinations of these compounds in biological and environmental samples. Rodil et al. [119] established a novel analytical method for the determination of UV sunscreen agents, including three highly polar sulfonates (e.g., 2-phenylbenzimidazole-5-sulfonic acid) and six other less polar compounds (e.g., benzophenone-3, octocrylene …) in water environments based on SPE and LC-ESI-MS^2^. Detection limits between 7 and 46 ng·L^−1^ were achieved. More recently, these authors developed a method to determine a group of 53 multiclass emerging organic pollutants (including the types previously mentioned) using LC-MS^2^ after SPE. The proposed method allowed LODs between 0.3 and 30 ng·L^−1^ [115].

Zhao et al. developed a method to determine triclosan and triclocarban in wastewater and tap-water samples. Enrichment of the target analytes before the analysis was performed using ionic liquid dispersive liquid-phase microextraction. The sensitivity of the proposed method allowed LODs in the range 0.04–0.58 *μ*g·L^−1^ [110]. Klein et al. determined triclocarban in wastewater effluents by LC-QqQ after stir-bar sorptive extraction (SBSE) and obtained a LOQ of 10 ng·L^−1^ for the target analyte [111].

Derivatives of 2-hydroxybenzophenone are extensively employed as UV absorbers. With respect to their toxicological effects, in vivo and in vitro studies have demonstrated that some hydroxylated benzophenones exhibit estrogenic and antiandrogenic activities [146]. Negreira et al. [112] determined six derivatives of 2-hydroxybenzophenone in water samples. The compounds were first concentrated using a solid-phase extraction (SPE) cartridge and were then selectively determined by liquid chromatography-tandem mass spectrometry (LC-MS^2^) using electrospray ionisation (ESI) in positive and negative modes, except for one compound (2-hydroxy-4-methoxybenzophenone-5-sulfonic acid) that could be ionised only in negative mode. The proposed method provided LOQs from less than 1 to 32 ng·L^−1^, depending on the compound and the type of water sample. Pedrouzo et al. [114] determined eleven PCPs, including hydroxylated benzophenones, triclocarban and triclosan, and parabens (another type of preservative used in personal care products) by SPE and UHPLC-MS^2^ in surface and wastewaters; the chromatographic separation required only 9 minutes. 

LC-MS^2^ with ESI operated in negative mode, and MRM was used in combination with liquid-liquid extraction by Zhang et al. [116] to analyse benzophenone UV filters in sediments and sludge. Their developed method allowed LOQs in the ranges of 0.06–0.33 ng·g^−1^ dry weight (dw) and 0.1–1.65 ng·g^−1^ dw for sediment and sludge samples, respectively. UHPLC-MS^2^ was used by Nieto et al. [118] for the determination of a group of parabens and two UV filters in sewage sludge after pressurised liquid extraction. The LODs and LOQs were less than 8 *μ*g·kg^−1^ and 12.5 *μ*g·kg^−1^ of dw, respectively.

The occurrence of some lipophilic UV filters in fish has been known for some time; recently, however, a method for the simultaneous determination of nine polar and lipophilic UV filters in fish was reported by Zenker et al. [120]. Mid-polar and lipophilic UV filters were extracted from homogenised tissue using a mixture of ethyl acetate, n-heptane, and water, followed by a clean-up with reversed-phase HPLC and HPLC-MS analysis. Recoveries of polar to lipophilic compounds from fish tissue exceeded 72% for all nine UV filters.

Calafat et al. [147] analysed urine samples using online solid-phase extraction coupled with isotope-dilution high-performance liquid chromatography/tandem mass spectrometry for the determination of four parabens that are widely used as antimicrobial preservatives. They studied the differences between the concentrations of parabens in urine samples according to sex and race/ethnicity to investigate differences in the use of personal care products that contain these compounds.

Kim and collaborators [121] developed a multiresidue analytical method for the determination of PCPs belonging to different classes (antimicrobials, preservatives, benzotriazole UV stabilisers (BUVSs) and organophosphorous compounds (OPCs)) in fish using high-speed solvent extraction (HSSE) followed by silica-gel clean-up and ultra-fast liquid chromatography coupled with tandem mass spectrometry (UFLC-MS^2^) analysis. The developed extraction and clean-up method resulted in a good recovery (>70%) for all the four groups of compounds, with RSDs that ranged from 0.7 to 15.4%.

### 4.3. Alkylphenol Polyethoxylates (APEOs) and Bisphenol A

Alkylphenol polyethoxylates (APEOs) are a class of non-ionic surfactants that are used extensively as detergents, emulsifiers, wetting agents, and dispersing agents in industrial, agricultural, and household applications. Because alkyl-substituted phenols are relatively polar substances with an –OH group, they are highly soluble in water, which increases their potential to pollute water. APEOs are known to be broken down in WWTPs, which leads to the formation of subproducts that are more toxic, lipophilic, estrogenic, and persistent than the parent substances. APEOs degrade to nonylphenols (NPs) or, to a lesser extent, octylphenols (OPs), which are considered persistent environmental pollutants. In the past 20 years, several groups of authors have reported that this class of compound exhibits bioaccumulation in aquatic organisms [148] and chronic toxicity [149] and can mimic natural hormones and disrupt endocrine functions by interacting with estrogen receptors [150–152].

Bisphenol A (BPA) is used extensively in the industrialised world and is present in a diverse range of manufactured products. It is a monomer used in the manufacture of epoxy, polycarbonate, and polyester styrene resins. Such resins are widely used in canned-food and beverage packaging and in dental resins, which leads to potential human exposure to BPA. The estrogenic properties of this compound have been demonstrated [153].

Although the conventional analytical methods used to extract APEO compounds from liquid samples were initially based on liquid-liquid extraction (LLE), researchers have recently favoured the replacement of LLE with SPE for liquid samples [152]. Loyo-Rosales et al. developed an offline SPE procedure to extract APs, short-chain APEOs (AP1-5EO) [154], and long-chain APEOs (AP6-16EO) [155] in water. Recoveries for the APs and short-chain APEOs were greater 81% for all of the analytes, with variations in response in the range 1–14% (RSD).

In addition, the use of more specific extraction techniques for APEOs and BPA has also been reported [152]. For example, several types of fibre have been tested for extraction of these substances by solid-phase microextraction (SPME) [156]. Thus, other more specific microextraction techniques, such as SBSE [157], LPME, and dispersive liquid-liquid microextraction (DLLME), have also been used [158].

Wang and Schnute [124] reported an ultra-high-performance liquid chromatography/tandem mass spectrometry (UHPLC/MS^2^) method without previous sample preparation for the simultaneous quantification of NP and BPA. All target analytes were chromatographically separated within 3 min and LODs in the range 0.04–0.057 *μ*g·L^−1^ were achieved.

Their physicochemical profiles suggest that some APEOs and degradation products have a strong affinity towards organic matter. They tend to bind to sediments and to accumulate in aquatic organisms due to their high lipophilicity and lower water solubilities [152]. Extraction from sediment can be performed by Soxhlet extraction, UAE, SFE, MAE, or PLE. Further purification of the extract obtained is usually performed by SPE [152, 159]. For example, Petrovic et al. [160] used PLE with acetone : methanol 1 : 1 (v : v) to extract alkylphenolic (AP) compounds from sludge and achieved recoveries of 78% for nonylphenoxy carboxylate (NPE1C), 68% for nonylphenoxy ethoxy carboxylate (NPE2C), and 81% for NP. Fountoulakis and collaborators [161] develop a microwave-assisted extraction method for the determination of the NP and NPEO in sewage sludge and compared this method with more traditional methods, such as Soxhlet extraction and sonication. The detection limit was 1.82 *μ*g·g^−1^ for NPEO and 2.86 *μ*g·g^−1^ for NP.

The number of papers reporting the use of MAE has therefore increased considerably [67]. Croce et al. [162] compared MAE with PLE for the isolation of NP and NPEOs from river sediments. They concluded that MAE has an important disadvantage compared to PLE extraction—the need for sample centrifugation and filtration—that can critically affect the analytical accuracy. However, this drawback can be overcome through the use of proper accessories for automatic sample handling. Moreover, MAE also offers the ability to extract several samples simultaneously, whereas, in PLE, samples are always run one at a time. MAE has therefore become the most suitable extraction system for monitoring programs because of its ability to handle a large number of samples in a short period of time [67, 152].

Dorival-García et al. [76] have presented a comparison of three extraction techniques—ultrasound-assisted extraction, microwave-assisted extraction, and pressurised liquid extraction—to evaluate their efficiency in the determination of bisphenol A and its chlorinated derivatives in sewage sludge samples. The statistical comparison of the methods demonstrated no statistically significant differences between the extraction techniques for the determination of BPA and chlorinated derivatives in sludge samples.

With respect to biota, only a few studies have been conducted. Tavazzi et al. [125] described a PLE method followed by LC-MS analysis for the determination of OP, NP, and BPA in fish liver. After the authors compared the efficiency of PLE with conventional Soxhlet extraction, they applied the developed procedure to the analysis of liver samples. The limits of detection (LOD) were 5 ng·g^−1^ for 4-t-octylphenol, 15 ng·g^−1^ for bisphenol A, and 20 ng·g^−1^ for nonylphenol. A high-speed and robust online SPE-HPLC-MS method was developed for the analysis of five estrogens and bisphenol A (BPA) in milk samples by Yan et al. [126], who used a triacontyl-bonded silica (C30) extraction column. This report represents the first report of a C30 online SPE-LC-MS analytical method for the screening and monitoring of these estrogens and BPA in milk samples. Large-volume injection (1 mL) could be achieved with this method; the recoveries for all of the analytes ranged from 71.4 to 97.1%, and the reproducibility was less than 15%. 

A rapid liquid chromatography-tandem mass spectrometry (LC-MS^2^) method was developed for the simultaneous analysis of nine bisphenol A ether derivatives [127]. The method was applied to the determination of these compounds in canned soft drinks and canned foods. OASIS HLB solid-phase extraction (SPE) cartridges were used for the analysis of soft drinks, whereas solid canned foods were extracted with ethyl acetate. The method limits of quantitation ranged from 0.13 *μ*g·L^−1^ to 1.6 *μ*g·L^−1^ for soft drinks and from 1 *μ*g·kg^−1^ to 4 *μ*g·kg^−1^ for food samples.

Table 3 shows diverse determinations of these compounds in biological and environmental samples.

### 4.4. Brominated Flame Retardants

The last group of selected compounds are brominated flame retardants. Brominated flame retardants include polybrominated diphenyl ethers (PBDEs), polybrominated biphenyls (PBBs), brominated cyclohydrocarbons, decabromodiphenyl ethers (DeBDEs), hexabromocyclododecanes (HBCDs), and tetrabromobisphenol A (TBBPA) [163–165]. Because of their widespread presence in the environment and their potential toxicity to humans and animals, increasing concern has prompted many countries to ban some of these compounds.

Brominated flame retardants have been used for many years in a variety of commercial products, including children's sleepwear, foam cushions in chairs, computers, plastics, and electronics, to keep them from catching fire. Brominated flame retardants work by releasing bromine free radicals when heated, and these free radicals scavenge other free radicals that are part of the flame propagation process. The use of these flame retardants is believed to have successfully reduced fire-related deaths, injuries, and property damage. However, their widespread presence in the environment and in human and wildlife samples, as well as their presence in locations far from where they were produced or used, has raised concerns. They are environmentally persistent and lipophilic, and they bioaccumulate in animals and in humans.

In 2004, the European Union banned the use of the penta- and octa-BDEs; later, in 2008, they also banned deca-BDEs. However, deca-BDEs are still being manufactured and used. Previous studies suggested that deca-BDE is too large to bioaccumulate and would not be a risk to humans. However, research now shows that it can accumulate in animal tissues (including those of humans) and that it can debrominate in the environment and metabolically form the lower-brominated species (including the octa- and penta-BDEs) [17]. 

Because of the hydrophobic character of PBDEs and their low concentration in water in liquid-liquid extraction, the use of large volumes as high 1000 mL is necessary. The most popular extractive solvents are hexane, isooctane, and tert-butyl ether [132]. However, this technique is often replaced by solid-phase extraction [131, 166]. Until recently, atmospheric-pressure photoionisation (APPI) has typically been used for the determination of nonpolar halogenated flame retardants (HFRs) by liquid chromatography-tandem mass spectrometry. However, atmospheric-pressure chemical ionisation (APCI) offers three advantages: simplicity, rapidity, and high sensitivity.

Bacaloni et al. described a liquid chromatography technique with negative-ion-atmospheric-pressure photoionization-tandem mass spectrometry (LC/NI-APPI-MS^2^) method for the simultaneous determination of TBBPA and five PBDEs in water samples. A mobile phase that consisted of methanol/acetone/water was used, where acetone served as the dopant for APPI. BDEs were poorly retained by solid-phase extraction (SPE) from river water and sewage treatment plant effluent; thus, liquid-liquid extraction (LLE) with n-hexane should be used for these samples. The recoveries of TBBP-A and PBDEs from tap water (SPE), river water, and industrial wastewater (LLE) were in the range of 81–88%, 78–92%, and 43–99%, respectively, with relative standard deviations less than 17% and LOQs of 0.2–3.3 ng·L^−1^, except for one compound [132]. 

Guerra et al. developed a method for the simultaneous determination of HBCD diastereoisomers and TBBPA and its derivatives using LC-QqLIT-MS [166]. Two different experiments were developed. The first experiment was based on a selected reaction monitoring (SRM) method; the second was based on an ion trap used for the storage and subsequent fragmentation of precursor ions, which resulted in an enhanced product-ion (EPI) experiment. 

Zhou et al. [131] studied the use of liquid chromatography with atmospheric-pressure chemical ionisation (APCI) tandem mass spectrometry (LC-APCI-MS^2^) for analysis of 38 HFRs from wastewater samples and found ng·L^−1^ levels. Compared with APPI, APCI does not require a UV lamp and a dopant reagent to assist the atmospheric-pressure ionisation. All the isomers and the isobaric compounds were well resolved within 14 minutes of LC separation time.

Ueno et al. developed a method for determining the occurrence of hydroxylated PBDEs (OH-PBDEs) (metabolites of PBDEs) in abiotic environments [167]. In this study, OH-PBDEs were determined in samples of surface water and precipitation (rain and snow) collected from sites in Ontario, Canada. OH-PBDEs were detected in all the samples analysed. The results in this study suggested that OH-PBDEs were ubiquitous in the abiotic environment and were most likely produced through reaction of PBDEs with atmospheric OH radicals. In addition, the authors speculated that they may be present in surface water near STPs due to oxidation of PBDEs and inflows from metabolism by humans and animals. 

Soxhlet extraction is a widely used standard technique for the determination of brominated flame retardants (BFRs) in sewage sludge. However, new extraction techniques (e.g., USE, MAE, and PLE) have also been evaluated.

Ruan et al. [168, 169] reported, for the first time, the discovery of a new class of brominated flame retardant, tris (2,3-dibromopropyl) isocyanurates, in the environment. These compounds were found in all of the river water, sediment, soil, and biota samples analysed from a factory-polluted area in southern China. The authors developed a high-performance liquid chromatography-tandem mass spectrometry (HPLC tandem MS) method. Water samples were extracted using a SPE process. Soils, surface sediments, earthworm, and tissues/organs of carp samples were extracted with dichloromethane (DCM) using accelerated solvent extractor (ASE). 

An LC-IT-MS method employing ESI operated in negative ionisation mode was developed to determine HBCD diastereoisomers in marine sediment samples; the LOQs ranged from 25 to 40 pg·g^−1^ (dw). Target analytes were extracted from sediment samples by MAE. The efficiency of this technique was compared with those of Soxhlet extraction and PLE, and the obtained results showed that MAE provides better extraction efficiencies than either PLE or Soxhlet extraction [134]. 

In another paper, the extraction efficiency of pressurised liquid extraction (PLE), microwave-assisted extraction (MAE), and ultrasonic-assisted extraction (UAE) under different conditions was compared for the recovery of the most commonly employed brominated flame retardants (BFRs) from styrenic polymeric matrixes. A HPLC-MS^2^ method combined with PLE resulted in complete extraction of TBBPA and HBCD (95–100% recovery) and intermediate recovery rates for deca-BDE (50%). The performance of MAE, however, was similar to that of PLE for HBCD, but lower extraction yields were achieved for TBBPA and mainly deca-BDE. Ultrasonication, in comparison, offered relatively low extraction recoveries (10–50%) [170].

To extract PBDEs from biological samples, multistage liquid-liquid extraction with solvents of diverse polarity is used. The preliminary process includes denaturation of the proteins in the samples. The main disadvantage of this process is often the long time required to reach division of phases or the necessity of sample centrifugation. The amount of solvents used can be reduced through the application of SPE and another extraction technique.

Gómara et al. [138] reported a method for the enantiomer-specific determination of HBCDs by LC-ESI-MS^2^ from food samples. Food samples, with the exception of butter, which was extracted by dialysis in n-hexane, were extracted by matrix solid-phase dispersion (MSPD) with an acetone : n-hexane mixture (50 : 50, v/v). The detection limits ranged from 1.5 to 4.3 ng·mL^−1^, and the repeatability and reproducibility, expressed as the relative standard deviation, were less than 6% and 13%, respectively.

A comprehensive, sensitive, and high-throughput liquid chromatography-atmospheric-pressure photoionisation tandem mass spectrometry (LC-APPI-MS^2^) method was developed for the analysis of 36 halogenated flame retardants (HFRs) from twenty-two fish samples. Automated pressurised liquid extraction (PLE) was applied. Under the optimised conditions, all of the HFRs were eluted from the LC column within 14 min. Excellent detection limits that averaged 4.7 pg·g^−1^ were obtained [139].

Tang developed a method for the simultaneous determination of three diastereoisomers of hexabromocyclododecane (HBCD) in human plasma using liquid chromatography-tandem mass spectrometry (LC-MS^2^). The simple pretreatment involved protein precipitation with methanol (MeOH). The recoveries ranged from 79.0% to 108.9%, and the LOQ was 10 pg·mL^−1^ for each diastereoisomer. No significant matrix effect or carryover effect of the analytes was observed in this study [171]. 

Table 4 shows diverse determinations of these compounds in biological and environmental samples.

## 5. Conclusions and Future Trends

Various types of natural and synthetic chemical compounds that mimic or inhibit the natural action of the endocrine system in animals and humans are defined as EDCs. The trace concentration levels of EDCs and their diversity in various aquatic environments have been recognised. One of the ongoing research trends concerning EDCs involves the identification and determination of their effects on both the environment and humans.

For the quantitative analysis of EDCs, mass-based analytical methods exhibit excellent sensitivity and precision for individual EDCs. The application of advanced LC-MS^2^ technologies to environmental and biological analyses has expanded the range of compounds that can be analysed, which has permitted more comprehensive assessments of environmental contaminants. In recent years, advanced instruments, such as LC-MS^2^, LC-TOF-MS, and LC-LIT-MS, have been developed and widely employed in the analysis of EDCs in environmental and biological matrices. These advanced instruments have been shown to be helpful in the quantification of trace levels of these compounds with high precision and sensitivity. In addition, these analyses processes typically require an extraction step prior to the instrumental procedure. The extraction step plays a key role in determining the overall level of analytical performances, and several techniques have been developed. With respect to the first steps of the analysis (i.e., the sample extraction and purification steps), an extensive amount of development has been performed to obtain greener methodologies, such as SPE, SPME, and LPME for liquid samples and USE, MAE, PLE for solid samples, that consume less solvent and energy. In addition, numerous improvements have been derived from the development of new materials for these first steps of the analysis. 

Additional research is clearly needed to determine the breakdown pathways and to evaluate the fate of the transformation products of EDCs. Therefore, the development of future generic analytical protocols should permit the simultaneous determination of parent compounds and their metabolites.

Numerous studies have shown that organic pollutants are incompletely eliminated during wastewater treatment processes; thus, the next task is to improve the treatment processes to remove a large number of very different micropollutants. The challenges include the identification of new emerging compounds, the establishment of appropriate standards, the development of strategies to reduce inputs to the aqueous environment, and the application of novel monitoring methods.

## Figures and Tables

**Table 1:** Determination of steroid hormones in biological and environmental samples.

Analytes	Samples	Extraction	Determination	Analytical parameters	Reference
26 steroids including natural and synthetic estrogens, progestogens, and androgens	Water samples	SPE	LC-MS^2^	Recovery > 80% MDLs: 0.1–0.73 ng·L^−1^	[105]
Resveratrol, daidzein, coumestrol, genistein	River water	SPE	LC- MS^2^	Recovery > 80% LODs ≤ 2 ng·L^−1^	[106]
36 endocrine disrupting chemicals including estrogens and progestogens	Potable and river water	SPE	UPLC-Q-TOF-MS	Recovery: 46–134% LODs < 0.72 ng·L^−1^	[107]
Boldenone, nandrolone, testosterone, methyltestosterone, epiandrosterone, androsterone, satnozolol	Human urine	In-tube SPME	LC-MS^2^	Recovery: 86–117% LODs: 9–182 pg·mL^−1^	[108]
28 steroids including natural and synthetic estrogens, androgens, progestogens, and glucocorticoids	Sludge	UAE	LC-MS^2^	Recovery: 63–138% LODs: 0.08–2.06 ng·g^−1^	[100]
*β*-Estradiol, estriol, 17*α*-ethinylestradiol	Sewage sludge	MAE	LC-MS^2^	Recovery: 72–103% LODs: 0.6–3.5 ng·g^−1^	[75]
Estrone, testosterone, androstenedione, norethindrone, levonorgestrel, progesterone	Soil	PLE	LC-MS^2^	Recovery: 45–100% LODs: 0.08–0.89 ng·g^−1^	[88]
*α*-Estradiol, *β*-estradiol, estriol, estrone and ethynylestradiol and their sulfate, glucuronide and acetate conjugates	River sediments	MAE	LC-MS^2^	Recovery: 83–107% LODs < 1 ng·g^−1^	[109]
Estrone, 17*α*-estradiol, 17*β*-estradiol, estriol, 17*α*-ethinylestradiol, diethylstilbestrol, estradiol 17-glucoronide, estrone 3-glucoronide, estradiol 3-sulfate, estrone 3-sulfate, estradiol 17-acetate	Sewage sludge	PLE	LC-MS^2^	Recovery > 81% LODs < 26 ng·g^−1 ^	[85]
Estradiol, estrone, estriol, estradiol-17-glucuronide, estrone-3-sulfate, ethynylestradiol, diethylstilbestrol, bisphenol, progesterone, levonorgestrel, norethindrone	River water	SPE	LC-MS^2^	Recovery: 70–104% MDLs ≤ 3.94 ng·L^−1^	[97]
Eighteen androgens and progestogens	Environmental waters	DLLME-SFO	UHPLC-MS^2^	Recovery: 87–116% LODs: 0.8–3.1 *μ*g·L^−1^	[99]
Flurogestone acetate, delmadinone acetate, megestrol acetate, chlormadinone acetate, melengestrol acetate, medroxyprogesterone acetate, and chlorotestosterone acetate	Kidney	ASE	LC-MS^2^	Recovery: 17–58%	[103]

LC: liquid chromatography; UHPLC: ultra high-pressure liquid chromatography; MS: mass spectrometry; TOF-MS: time-of-flight mass spectrometry.

LOQ: limit of quantification, LOD: limit of detection; MDL: method detection limit.

SPE: solid-phase extraction; SPMe: solid-phase microextraction; UAE: ultrasound-assisted extraction; PLE: pressurised liquid extraction; MAE: microwave-assisted extraction; ASE: accelerated solvent extraction; DLLME-SFO: dispersive liquid-liquid microextraction method based on the solidification of a floating organic drop.

**Table 2:** Determination of PCPs in biological and environmental samples.

Analytes	Samples	Extraction	Determination	Analytical parameters	Reference
Triclosan and triclocarban	Wastewater and tap water	IL-DLLME	LC-MS^2^	Recovery: 70–103% LODs: 0.04–0.58 *μ*g·L^−1^	[110]
Triclocarban	Wastewater effluents	SBSE	LC-MS^2^	Recovery: 92–96% LOQ: 10 ng·L^−1^	[111]
UV filters: BP-1, BP-2, BP-3, BP-4, BP-6, and BP-8	River water and wastewater	SPE	LC-MS^2^	Recovery: 83–105% LOQs: 1–32 ng·L^−1^	[112]
Benzotriazoles (UVP, UV 329, UV 326, UV 328, UV 327, UV 571, UV 360)	Coastal marine water and wastewater	On-line SPE	UHPLC-MS^2^	Recovery: 65–94% LODs: 0.6–4.1 ng·L^−1^	[113]
BP-1, BP-3, BP-8, OC, OD-PABA Triclocarban, triclosan Methylparaben, ethylparaben, benzylparaben, propylparaben	Surface water and wastewaters	SPE	UHPLC-MS^2^	Recovery: 20–101% LODs: 20–200 ng·L^−1^	[114]
53 multiclass emerging pollutants (UV filters and insect repellents, among others)	Tap water, surface water and wastewater	SPE	LC-MS^2^	Recovery > 60% LODs: 0.3–30 ng·L^−1^	[115]
4-hydroxybenzophenone BP-1, BP-2, BP-3, and BP-8	Sediments and sludge	LLE	LC-MS^2^	Recovery: 70–116% LOQs: 0.06–1.65 ng·g^−1^	[116]
Biocides, UV filters, and benzothiazoles	Sludge sample	PLE	LC-MS^2^	Recovery: 74–119%	[117]
Triclosan and triclocarban methyl paraben, ethyl paraben, propyl paraben, and benzyl paraben OD-PABA, OC, PMDSA, BP-1, BP-3, BP-8	Sewage sludge	PLE	UHPLC-MS^2^	Recovery: 15–100% LODs < 8 ng·g^−1^	[118]
PBSA, BP-3, OC, OD-PABA, BP-4, 4-MBC, BM-DBM, PDT, and IAMC	Water environment	SPE	LC-MS^2^	Recovery: 63–102% LODs: 7–46 ng·L^−1^	[119]
Benzophenone-1, benzophenone-2, benzophenone-3, benzophenone-4, 4,4-dihydroxybenzophenone, ethyl-4-aminobenzoate, 2-ethylhexyl-4-trimethoxycinnamate, 3-(4-methylbenzylidene)-camphor, 3-benzylidene-camphor	Fish	Ethyl acetate, n-heptane, and water	LC-MS	Recovery > 72% LODs: 78–205 ng·g^−1^ 1.8–5.3 *μ*g·kg^−1^	[120]
Antimicrobials, preservatives, and benzotriazole UV stabilizers	Fish	HSSE	UFLC-MS^2^	Recovery > 70% MDLs: 179–266 ng·g^−1^	[121]

LC: liquid chromatography; UHPLC: ultra high-pressure liquid chromatography; UFLC: ultra fast liquid chromatography; QqQ: triple quadrupole; IT: ion trap; LIT: lineal ion trap; ESI: electrospray ionization; APCI: atmospheric pressure chemical ionization; NI: negative ion mode of ionisation; PI: positive ion mode of ionisation; MRM: multiple-reaction monitoring; SRM: selected reaction monitoring.

IL: ionic liquid; LLE: liquid-liquid extraction; SDME: single drop microextraction; DLLME: dispersive liquid-liquid microextraction; SBSE: stir bar sorptive extraction; MISPE: molecularly imprinted polymer extraction; UAE: ultrasound-assisted extraction; PLE: pressurised liquid extraction; MAE: microwave-assisted extraction; HSSE: high-speed solvent extraction.

LOQ: limit of quantification; LOD: limit of detection; MDL: method detection limit.

OC: octocrylene; PMDSA: 2-phenylbenzimidazole-5-sulfonic acid; OD-PABA: octyldimethyl-p-aminobenzoic acid; BP-1: 2,4-dihydroxybenzophenone; BP-2: 2,2′,4,4′-tetrahydroxybenzophenone; BP-3: 2-hydroxy-4-methoxybenzophenone; BP-4: 2-hydroxy-4-methoxybenzophenone-5-sulphonic acid; BP-6: 2,2′-dihydroxy-4,4′-methoxybenzophenone; BP-8: 2,2′-dihydroxy-4-methoxybenzophenone; BM-DBM: butylmethoxydibenzoylmethane; IAMC: isoamyl methoxycinnamate; 4-MBC: 3-methylbenzylidene camphor; PDT: phenyldibenzimidazoletetrasulfonic acid.

**Table 3:** Determination of alquilphenols in biological and environmental samples.

Analytes	Samples	Extraction	Determination	Analytical parameters	Reference
Octyl, nonylphenol ethoxylates, and carboxylates	Wastewater	SPE, LLE	LC-MS^2^	Recovery: 21–71% LODs: 2–29 ng·L^−1^	[122]
APs, APEOs, APECs	Surface, wastewater	SPE	LC-MS^2^	Recovery: 50–90% LODs: 1–100 ng·L^−1^	[123]
OP, NP, and BPA	Bottled water	—	UHPLC-MS^2^	Recovery: 97–106% LODs: 0.04–0.057 *µ*g·L^−1^	[124]
4-t-Octylphenol, 4-nonylphenols, and bisphenol A	Fish liver	ASE	LC-MS	Recovery: 53% LODs: 5–20 ng·g^−1^	[125]
Bisphenol A	Bovine milk	SPE	LC-MS	Recovery: 71–97% LOD: 0.20 *μ*g·L^−1^	[126]
Bisphenol A-diglycidyl ether, bisphenol F-diglycidyl ether, and their derivatives	Canned food and beverages	SPE	LC-MS^2^	Recovery: 60–95% MQLs: 0.13–0.6 *μ*g·L^−1^ MQLs: 1–4 *μ*g·kg^−1^	[127]
Nonionic surfactants	Wastewater	—	LC-QTOF-MS	MDLs: 10–200 *µ*g·L^−1^	[128]
APs, AP1-15EOs	Amended soil	PLE	LC-MS	Recovery: 36–110% LODs: 0.3–30 ng·g^−1^	[129]
4-Tert-octylphenol, 4-octylphenol, 4-n-nonylphenol, nonylphenol, and bisphenol A	Sea water	DLLME	LC-MS^2^	Recovery: 90–108% MQLs: 0.005–0.03 *μ*g·L^−1^	[130]

LC: liquid chromatography; UHPLC: Ultra high-pressure liquid chromatography; TOF: time-of-flight; MS: mass spectrometry.

SPE: solid-phase extraction; LLE: liquid-liquid extraction; PLE: pressurised liquid extraction; ASE: accelerated solvent extraction; DLLME: dispersive liquid-liquid extraction.

MQL: method quantification limit; LOQ: limit of quantification; LOD: limit of detection; IDL: instrument detection limit.

BPA: bisphenol A; NP: nonylphenol; OP: octylphenol; APs: alkylphenols; APEOs: alkylphenol polyethoxylates; OPEOs: octylphenol ethoxylates

PE1C: nonylphenoxy carboxylate; NPE2C: nonylphenoxy ethoxy carboxylate.

**Table 4:** Determination of flame retardants in biological and environmental samples.

Analytes	Samples	Extraction	Determination	Analytical parameters	Reference
38 HFRs	Wastewater	Preconditioned empire speed disk	LC–MS^2^	Recoveries: 25–132% LOQs: 0.1–5.6 *μ*g·L^−1^	[131]
TBBPA and five PBDEs	Wastewater, river, and drinkingwater	LLE	LC-LIT	Recoveries: 43–99% LOQs: 0.2–3.3 ng·L^−1^	[132]
HBCD isomers	Suspended sediments from detroit river	ASE	LC-MS^2^	—	[133]
HBCD diastereoisomers	Marine sediment	MAE	LC-IT	Recoveries: 68–91% LOQs: 25–40 pg·g^−1^	[134]
TBBPA and brominated BPA analogues	Sediment and sludge	Soxhlet	LC-MS^2^	Recoveries: 70–105% LOQs: 0.02–0.15 ng·g^−1^	[135]
TBBPA, BPA, MonoBBPA, DiBBPA, and TriBBPA	Sewage sludge and sediment	Sonication SPE	LC-MS-LIT	Recoveries: 39–120% LODs: 0.6–2.7 ng·g^−1^	[136]
HBCDs and TBBPA	Sewage sludge	PLE	UHPLC-MS^2^	Recoveries: 65–112% LOQs: 0.005–0.14 ng·g^−1^	[137]
HBCDs (*α*-, *β*-, and *γ*-HBCD)	Food	MSPD	LC-MS^2^	LODs: 1.5–4.3 ng·mL^−1^	[138]
36 halogenated flame retardants	Fish	PLE	LC-MS^2^	IDLs: 4.7 pg	[139]

LC: liquid chromatography; UHPLC: ultra high-pressure liquid chromatography; MS: mass spectrometry; IT: ion trap; LIT: lineal ion trap.

SPE: solid-phase extraction; ASE: assisted solvent extraction; LLE: liquid-liquid extraction; PLE: pressurised liquid extraction; MAE: microwave-assisted extraction; MSPD: matrix solid-phase dispersion.

LOQ: limit of quantification; LOD: limit of detection; IDL: instrument detection limit.

HFRs: halogenated flame retardants; BPA: bisphenol A; TBBPA: tetrabromobisphenol A; PBDEs: polybrominated dipheny lethers; HBCD: hexabromocyclododecanes.
